# Regulation of TAZ in cancer

**DOI:** 10.1007/s13238-016-0288-z

**Published:** 2016-07-14

**Authors:** Xin Zhou, Qun-Ying Lei

**Affiliations:** 1 Key Laboratory of Metabolism and Molecular Medicine, Ministry of Education, and Department of Biochemistry and Molecular Biology and Institutes of Biomedical Sciences, Fudan University Shanghai Medical College, Shanghai, 200032 China; 2 Department of Cancer Biology, University of Texas MD Anderson Cancer Center, Houston, TX 77030 USA

**Keywords:** TAZ, the Hippo pathway, cancer

## Abstract

TAZ, a transcriptional coactivator with PDZ-binding motif, is encoded by *WWTR1* gene (WW domain containing transcription regulator 1). TAZ is tightly regulated in the hippo pathway-dependent and -independent manner in response to a wide range of extracellular and intrinsic signals, including cell density, cell polarity, F-actin related mechanical stress, ligands of G protein-coupled receptors (GPCRs), cellular energy status, hypoxia and osmotic stress. Besides its role in normal tissue development, TAZ plays critical roles in cell proliferation, differentiation, apoptosis, migration, invasion, epithelial-mesenchymal transition (EMT), and stemness in multiple human cancers. We discuss here the regulators and regulation of TAZ. We also highlight the tumorigenic roles of TAZ and its potential therapeutic impact in human cancers.

## INTRODUCTION

TAZ (Transcriptional coactivator with PDZ-binding motif), also known as WW domain containing transcription regulator 1 (*WWTR1*), was firstly identified as a 14-3-3 binding phosphoprotein (Kanai et al., 2000). TAZ is not a transcription factor because of lacking a DNA-binding domain, while TAZ could serve as a transcriptional regulator via its intrinsic transactivation domain (Kanai et al., 2000). TAZ has been implicated to interact with multiple transcription factors including RUNX2 (runt-related transcription factor 2) (Cui et al., 2003; Hong et al., 2005), MyoD (myoblast determination protein 1) (Jeong et al., 2010), PPAR (peroxisome proliferator-activated receptor) (Hong et al., 2005), TTF1 (thyroid transcription factor 1) (Di Palma et al., 2009; Park et al., 2004), PAX3 (paired box gene 3) (Murakami et al., 2006), PAX8 (paired box gene 8) (Di Palma et al., 2009), Smads (Varelas et al., 2008; Varelas et al., 2010), and TEADs (TEA domain transcription factors) (Mahoney et al., 2005; Zhang et al., 2009). Collaborating with these transcription factors, TAZ plays important roles during osteoblastic, myogenic, adipogenic differentiation (Hong & Yaffe, 2006). Although TAZ mainly functions as a transcriptional coactivator, recent studies have also elucidated that TAZ serves as a transcriptional repressor (Kim et al., 2015; Valencia-Sama et al., 2015). Since TAZ was firstly identified as a 14-3-3 binding protein, the mechanism for the specific regulation of TAZ was uncovered until 2008. Lei et al. demonstrated that TAZ is tightly regulated by the Hippo signaling pathway. Phosphorylation of TAZ at serine 89 is required for its interaction with 14-3-3 and substantial sequestration in the cytoplasm (Lei et al., 2008). Besides the 14-3-3 binding motif, WW domain, coiled-coil domain, and PDZ-binding motif within TAZ have important functions for the interaction with other partners of TAZ as well (Chan et al., 2011; Remue et al., 2010). TAZ-deficient mice develop significant renal cyst as early as embryonic day 15.5. Three weeks after birth, only one-fifth of TAZ-deficient mice are alive with dilated calyces, multiple renal cysts, and lung emphysema (Hossain et al., 2007; Makita et al., 2008; Tian et al., 2007). Till now, the *in vivo* pathogenic mechanism of TAZ has not been fully uncovered (Tian et al., 2007). Accumulating studies indicated that TAZ is an oncogenic protein during tumorigenesis via promoting cell proliferation, migration, and EMT (Chan et al., 2008; Chan et al., 2009; Lei et al., 2008; Zhang et al., 2009). Consistently, the expression of TAZ is elevated in multiple human cancers, such as invasive ductal breast cancer and glioblastoma (Bhat et al., 2011; Chan et al., 2008; Cordenonsi et al., 2011; Zhou et al., 2015a), implying the oncogenic roles of TAZ in human cancer development. Here we summarize the current understanding of the biochemical regulation of TAZ, highlight the intrinsic and extracellular signals that modulating TAZ activity, and emphasize the relevance of TAZ and human cancers.

## REGULATORY MECHANISMS FOR TAZ

As a transcriptional coactivator, TAZ is tightly regulated at certain layers. TAZ could be directly phosphorylated by LATS1/2 (the Hippo signaling), Cdk1, GSK3 or c-Abl in different circumstances; subcellular localization of TAZ is controlled by the Hippo signaling pathway in response to a bench of signals; LATS1/2, CK1, and GSK3 contribute to the protein stability regulation of TAZ; a negative feedback loop exists between TAZ and YAP; and the expression of TAZ is modulated by different miRNAs in cancer cells. Disturbance of any of these regulations may contribute to the oncogenic roles of TAZ in human cancer.

### Direct phosphorylation of TAZ

#### The Hippo pathway

**Figure 1:**
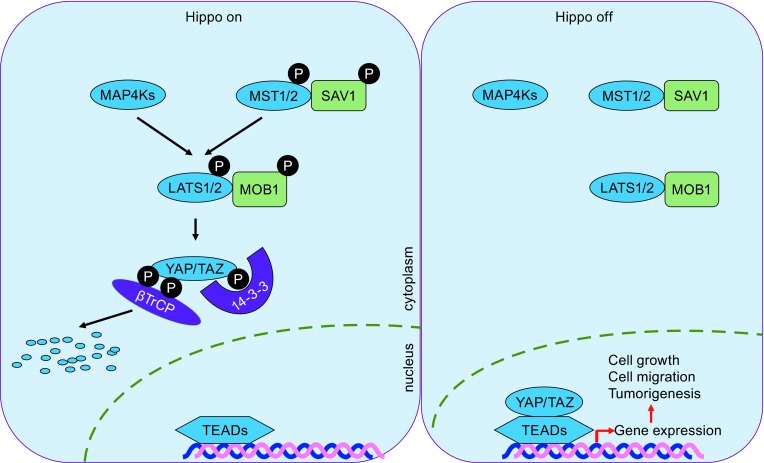
**The core of the Hippo signaling pathway**. The core components of the Hippo pathway comprise a kinase cascade and a transcriptional activation module. When the Hippo pathway is activated, mammalian STE20-like 1/2 (MST1/2) phosphorylate and form complex with Salvador 1 (SAV1). MST1/2 phosphorylate and activate large tumor suppressor 1/2 (LATS1/2) and Mob1 homolog (MOB1). Beside MST1/2, MAP4K family members could directly phosphorylate LATS1/2 as well. Two homologues transcriptional coactivator yes-associated protein (YAP) and WW domain-containing transcription factor (TAZ) are phosphorylated and inactivated by LATS1/2 via cytoplasmic retardation by 14-3-3 or degradation by SCF/CRL1(β-TrCP) E3 ligase. When the Hippo pathway is inactivated, MST1/2 and LATS1/2 are dephosphorylated and inactivated, resulting in the dephosphorylation and nuclear localization of YAP/TAZ. As transcriptional coactivators, the nuclear YAP/TAZ bind to and activate TEA domain family members 1–4 (TEAD1–4), leading to the transcription of genes relating to cell proliferation, migration, and tumorigenesis

The Hippo pathway plays a crucial role in organ size control and is highly conserved from *Drosophila* to mammals. TAZ and YAP (yes-associated protein), two mammalian homologous to *Drosophila* Yorkie, serve as two core downstream effectors of the Hippo signaling pathway which comprises an upstream kinase cascade and a downstream transcription module (Fig. 1). In a classical view, MST1/2, in complex with the non-catalytic partner SAV1, phosphorylate and activate LATS1/2, the NDR-family kinases. LATS1/2 are fully activated by MOB1 and then phosphorylate the downstream targets, i.e., YAP and TAZ (Bothos et al., 2005; Chan et al., 2005; Harvey & Tapon, 2007; Hergovich, 2011; Hergovich et al., 2006; Hoa et al., 2016; Zhao et al., 2010a). Although LATS1/2 are the kinases of TAZ, the requirement of MST1/2 for the phosphorylation and activation of LATS1/2 is cell type- and context-dependent. Recently, another pathway activating LATS1/2 was identified (Li et al., 2014; Meng et al., 2015; Zheng et al., 2015). Three groups independently found that MAP4K family members—Drosophila Happyhour (Hppy) homologues MAP4K1/2/3 and Misshapen homologues MAP4K4/6/7—serve as evolutionarily conserved direct kinases of LATS1/2 (Li et al., 2014; Meng et al., 2015; Zheng et al., 2015). Deletion of both MST1/2 and MAP4Ks but neither alone abolishes the phosphorylation of LATS1/2 (and thereby phosphorylation and inactivation of YAP/TAZ) in response to a wide range of upstream signals. Taken together, MST1/2 and MAP4Ks are two major kinase families, which can phosphorylate LATS1/2 directly upon multiple signals, to modulate the activity of TAZ. Elucidating the context dependency of MAP4Ks and MST1/2 will shed light on the regulation of TAZ.

When serine 89 of TAZ is phosphorylated by LATS1/2, TAZ is sequestrated in the cytoplasm by 14-3-3 to inhibit its nuclear functions (Lei et al., 2008). Multiple intrinsic and extracellular signals can inhibit the signaling cascade of the Hippo pathway, leading to the dephosphorylation and nuclear localization of TAZ (Liu et al., 2011; Yu et al., 2015). TEAD family transcription factors are the major binding partner of TAZ in the nucleus. The dephosphorylated TAZ interacts with TEADs and serves as a transcriptional coactivator to regulate the expression of a set of genes regarding cell proliferation, migration, and apoptosis (Zhang et al., 2009). It’s not clear whether the interaction between TAZ and other transcription factors would be regulated by the Hippo pathway.

Numerous *in vitro* studies identified that TAZ serves as an oncoprotein in cancer cells by supporting cell proliferation, migration, and EMT (Chan et al., 2008; Lei et al., 2008). While a rare mutation of *WWTR1*, which encodes TAZ protein, was observed in human cancer specimens (Gao et al., 2013). Interestingly, the expression level of TAZ protein was elevated in high-grade metastatic breast cancers (Chan et al., 2008; Cordenonsi et al., 2011). Several studies on the regulation of TAZ stability provided the explanation, at least partially, for the elevated TAZ level in human cancers.

It was believed the major regulatory mechanism of the Hippo pathway on TAZ is via modulating TAZ translocalization between cytoplasm and nucleus (Lei et al., 2008). Until 2010, Liu et al. uncovered that TAZ is a very unstable protein and degraded in cells under high cell density, and the stability of TAZ is regulated by the Hippo pathway, too (Liu et al., 2010). In response to high cell density, the Hippo pathway is highly activated and TAZ is primarily phosphorylated at serine 311 by LATS1/2 and further phosphorylated by CK1 at serine 314. The phosphorylated TAZ creates a phosphodegron for the recognition by the F-box protein β-TrCP. As a component of E3 ligase complex, β-TrCP recruits TAZ to the SCF/CRL1(β-TrCP) E3 ligase to carry on the polyubiquitylation and degradation (Liu et al., 2010). These studies suggest that TAZ could be regulated by the Hippo pathway via two distinct mechanisms, i.e. localization and stability (Fig. 1).

#### The AKT/GSK3 pathway

During cell contact inhibition, the Hippo pathway will be activated and then phosphorylated TAZ at serine 311 for further degradation by SCF/CRL1(β-TrCP) E3 ligase (Liu et al., 2010). Compared with YAP, TAZ has two phosphodegrons which can be recognized by β-TrCP (Huang et al., 2012; Liu et al., 2010; Zhao et al., 2010b). Besides the C-terminal phosphorylation of TAZ by LAT1/2, Huang and colleagues showed that the N-terminal of TAZ is phosphorylated by GSK3β at serine 58 and serine 62 directly. The phosphorylated serine 58 and serine 62 on TAZ create an N-terminal phosphodegron for β-TrCP recognition, interaction and further polyubiquitylation and degradation of TAZ (Huang et al., 2012). They further provided evidence that the N-terminal phosphorylation of TAZ is regulated by the PTEN/PI3K/AKT pathway but independent of the Hippo signaling pathway. Moreover, they observed that the protein level of TAZ correlates with the PI3K signaling activity, i.e., TAZ is elevated in PTEN mutant cancer cells (Huang et al., 2012). As we know, the PI3K/AKT pathway is frequently altered in multiple cancer types (Luo et al., 2003; Yuan & Cantley, 2008). This novel mechanism for TAZ stability regulation indicates that TAZ might be a crucial oncoprotein which acts downstream of the PI3K/AKT pathway during tumorigenesis. Taken together, the N-terminal and C-terminal phosphodegrons of TAZ which are responsible for the stability regulation of TAZ are differently modulated by the PI3K/AKT and the Hippo signaling pathways, suggesting that the protein level of TAZ can be manipulated in response to a wide range of intrinsic and extracellular signals through these two pathways. Recently, Feng et al. found that there is increased protein level and nuclear accumulation of TAZ during LPL1-induced osteogenic differentiation (Feng et al., 2015). Interestingly, this phenomenon relies on the AKT/GSK3β pathway but not LATS1/2 to stabilize TAZ. It’s well known that TAZ plays an important role during mesenchymal stem cell differentiation (Hong & Yaffe, 2006). However, how TAZ is regulated during differentiation remains unclear. Further studies will uncover the regulation of the Hippo pathway and the AKT/GSK3β pathway on TAZ during differentiation stages. Elucidating the contribution and collaboration of these two distinct pathways on TAZ regulation will provide novel insight into the elevated TAZ level in human cancer.

#### Cdk1

Besides LATS1/2 and GSK3, a new layer of phosphorylation regulation on TAZ has been identified recently from two independent groups (Zhang et al., 2015b; Zhao & Yang, 2015). Zhang and colleagues showed that the phosphorylation status of TAZ is changed during antimitotic drug-induced G_2_/M arrest. *In vitro* and *in vivo* studies demonstrated that TAZ is phosphorylated by the mitotic kinase cyclin-dependent kinase 1 (Cdk1) at serine 90, serine 105, threonine 326, and threonine 346 during the G_2_/M phase of the cell cycle. The mitotically phosphorylated TAZ is more oncogenic with stronger transcriptional activity. What’s more, if these sites cannot be properly phosphorylated by Cdk1, mitotic defects can be induced in MCF10A cells (Zhang et al., 2015b). Similarly, Zhao et al. observed that Taxol-induced TAZ phosphorylation and degradation is not dependent on the Hippo pathway (Zhao & Yang, 2015). Further studies showed that Cdk1 directly phosphorylates TAZ on six novel sites (serine 90, serine 105, threonine 175, threonine 285, threonine 326, and threonine 346). The phosphorylated TAZ is quite unstable in response to Taxol treatment, leading to the abolishment of TAZ-induced anti-tubulin drug resistance. In contrast, the dephosphorylation-mimicking mutant of TAZ is resistant to both Taxol and Vinblastine (Zhao & Yang, 2015). This study not only provides a novel kinase of TAZ, it also suggests that Cdk1-TAZ signaling plays a crucial role in anti-tubulin drug resistance in cancer cells. As we can compare, the phosphorylation sites from these two studies are almost the same, however, most of these sites are not conserved from YAP (Bui et al., 2016; Yang et al., 2013). YAP and TAZ share the similar regulatory mechanism under the modulation of the Hippo signaling pathway. It’s very interesting to identify the different regulatory mechanism of TAZ by other kinases, such as Cdk1.

As we know, SCF/CRL1(β-TrCP) is the *bona fide* E3 ligase for TAZ degradation. However, disruption of both phosphodegrons for β-TrCP recognition on TAZ doesn’t significantly rescue Taxol-induced TAZ degradation, and the interaction between TAZ and β-TRCP is independent of Cdk1 phosphorylation. These data strongly imply that the E3 ligase which is responsible for TAZ degradation upon Taxol treatment may not be β-TRCP. It’s intriguing to identify the novel E3 ligase which is responsible for Taxol-induced TAZ degradation by screening a pool of E3 ligase siRNA library.

#### c-Abl

In contrast to the phosphorylation of TAZ on serine or threonine, tyrosine phosphorylation also exists on TAZ. A recent study showed that TAZ undergoes tyrosine phosphorylation at tyrosine 316 by c-Abl kinase under hyperosmotic stress. The tyrosine phosphorylated TAZ selectively binds to nuclear factor of activated T cells 5 (NFAT5) and thereby inhibits its DNA-binding and transcriptional activity (Jang et al., 2012). This finding not only raised a novel type of phosphorylation on TAZ but also provided an intriguing hypothesis that TAZ can function as a transcriptional co-repressor of NFAT5 in renal cells under hypertonic condition. The TAZ-NFAT5 axis may provide a potential explanation for the TAZ deficiency-induced multiple kidney cysts (Hossain et al., 2007; Makita et al., 2008). The physiological significance of this phenomenon remains to be explored by future work.

### Feedback regulation from YAP

Intrinsic negative feedback loop is a broadly observed phenomenon in cells and plays important roles in maintaining homeostasis. This is true for YAP/TAZ feedback regulation. Moroishi et al. showed that elevated expression of YAP leads to the decrease of TAZ protein level in both cultured cells and mouse tissues. This phenomenon is dependent on TEADs-induced transcription of LATS1/2 and NF2 (neurofibromin 2), leading to the increase of both protein level and activity of LATS1/2 and thereby inhibition and degradation of TAZ (Moroishi et al., 2015). Interestingly, the stability of YAP is also regulated by TAZ in a similar negative feedback loop manner (Moroishi et al., 2015). This smart negative feedback loop establishes an efficient way to maintain the homeostasis of YAP/TAZ levels within cells. Similarly, Finch-Edmondson and colleagues found that TAZ protein accumulation is negatively regulated by YAP abundance in multiple mammalian cells (Finch-Edmondson et al., 2015). Both studies showed that this negative feedback loop is dependent on TEADs and LATS1/2. However, there are 2 major differences between Finch-Edmondson’s and Moroishi’s work: 1) Finch-Edmondson’s data showed that this negative feedback loop is uni-directional, i.e. only YAP expression level regulates TAZ degradation, but not in a reverse way; 2) Moroishi’s data support this feedback loop totally relies on LATS1/2, suggesting that YAP-induced TAZ reduction acts through LATS1/2 mediated degradation of TAZ via its C-terminal phosphodegron; while Finch-Edmondson implied that GSK3 but not CK1 is required for YAP-induced TAZ degradation, supporting that the N-terminal phosphodegron of TAZ may contribute to this specific modulation. These two studies identified novel regulatory mechanisms that maintaining YAP/TAZ at a constant level. Disruption of this homeostasis may be involved in tumorigenesis.

### Regulation of TAZ by microRNA

As we know from the well-established Hippo signaling pathway, the activity of TAZ is tightly regulated by phosphorylation from LATS1/2 (Yu et al., 2015; Zhao et al., 2008). However, more and more studies from clinic specimens imply that the mRNA level of TAZ is upregulated in these cancer samples, at least partially, because of the modulation from miRNAs. Yuan et al. found that miR-125a-5p is reversely correlated with TAZ in multiple glioma cell lines by targeting 3′UTR of TAZ mRNA directly and promoting its degradation. Furthermore, miR-125a-5p-induced cell growth inhibition and differentiation could be rescued by TAZ overexpression (Yuan et al., 2015). Recently, Li et al. reported that miR-125b directly targets TAZ by binding to its 3′UTR. Moreover, overexpression of TAZ impairs the miR-125b-induced inhibitory effect on growth and invasion of HCC cells (Li et al., 2015a). Based on a cancer-related miRNA screening in HCC cell lines, Higashi and colleagues showed that the mRNA level of miR-9-3p is inversely correlated with TAZ in HCC patients. However, whether TAZ serves as a major downstream target of miR-9-3p which inhibits HCC cell proliferation remains elusive (Higashi et al., 2015). Zuo and colleagues demonstrated that TAZ is one of the direct targets of miR-141 which was significantly decreased in gastric cancer. The growth inhibitory effect of miR-141 in gastric cancer cells dependents on TAZ (Zuo et al., 2015). In ovarian cancer cells, TAZ is validated as a direct target of miR-129-5p which plays a tumor-suppressive role in ovarian cancer. The inverse correlation between TAZ and miR-129-5p in ovarian cancer samples indicates that this regulation exists *in vivo* as well (Tan et al., 2015). Besides these miRNAs which directly bind to the mRNA of TAZ, other miRNAs which can modulate the upstream components of the Hippo signaling pathway are also involved in the activation of TAZ. For example, miR-130b directly represses MST1 and SAV1 expression in human glioblastoma cells and hence activates TAZ and TEAD transcription activity (Zhu et al., 2015). Interestingly, a recent study by Mori and colleagues uncovered that YAP/TAZ regulates cell-density-dependent global miRNA biogenesis by modulating the microprocessor machinery (Mori et al., 2014), implying a novel mechanism of YAP/TAZ in controlling cell growth and cancer. Taken together, YAP/TAZ are regulated by miRNAs and can regulate miRNA processing, leading us to consider whether a feedback loop exists between YAP/TAZ and miRNAs. Actually, Shen et al. provided an example for the feedback loop. One of the direct YAP targets, miR-130a, could strongly repress the inhibitory effect of VGLL4 on YAP, leading to the constitutive activation of YAP (Shen et al., 2015). The feedback loop between miRNA and YAP/TAZ might be involved in tissue homeostasis and cancer development.

## **INTRINSIC AND EXTRACELLULAR SIGNALS MODULATING TAZ**

**Figure 2:**
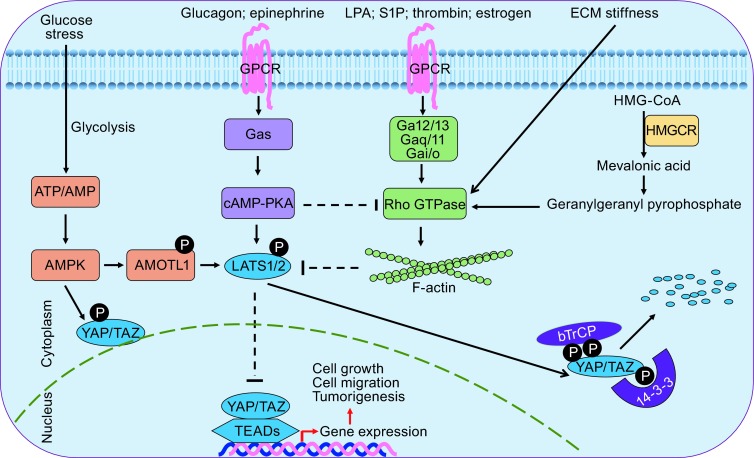
**Regulation of the Hippo pathway by multiple upstream signals**. Gαq/11-, Gα12/13-, and Gαi/o-coupled GPCRs signals activate YAP/TAZ via promoting Rho GTPase activation and cytoskeleton assembling, leading to the inhibition of LATS1/2 kinase activity; while Gαs-coupled GPCRs exert opposing roles on YAP/TAZ activation. The mevalonate cholesterol biosynthesis pathway, which is required for membrane localization and activation of Rho GTPase, promotes YAP/TAZ nuclear localization and activation. Inhibition of the mevalonate pathway is a potential way to target YAP/TAZ in human cancer. Under glucose deprivation condition, AMPK is activated to inhibit YAP/TAZ via direct phosphorylation on YAP/TAZ or via AMOTL-LATS1/2 axis

Extensive studies have identified numerous intrinsic and extracellular signals stimulating the Hippo signaling pathway, including contact inhibition, mechanic stress, cell junction, cytoskeletal rearrangement, cellular energy status, and the ligands stimulating GPCRs (Hansen et al., 2015; Yu et al., 2015) (Fig. 2). Other signals, such as hypoxia and osmotic stress, could modulate TAZ in a way independent of the Hippo signaling pathway (Hansen et al., 2015; Yu et al., 2015). The various stimuli on TAZ make it quite dynamic. How these signals are integrated to regulate TAZ under complex circumstances is very intriguing.

### Actin cytoskeleton integrates signals

Basically, the actin cytoskeleton and Rho-ROCK are important for maintaining the cell morphology and regulating cell proliferation and differentiation (Jaffe and Hall, 2005). Recent studies demonstrated that Rho GTPase and the dynamic of actin cytoskeleton play a central role in the regulation of the Hippo-YAP/TAZ signaling pathway (Fig. 2). 1) Multiple ligands of GPCRs could active/inactive TAZ via regulating Rho GTPase and actin cytoskeleton arrangement (Miller et al., 2012; Mo et al., 2012; Yu et al., 2012; Zhou et al., 2015a); 2) Overexpression of a constitutively active form of Rho GTPase or inhibition of Rho GTPase by C3 toxin strongly modulates the activity of TAZ (Dupont et al., 2011; Yu et al., 2012; Zhao et al., 2012). 3) Cell detachment inactivates TAZ via regulating cytoskeleton organization (Zhao et al., 2012); 4) Mechanical cues exerted by extracellular matrix stiffness and cell shape modulate TAZ activity, and this phenomenon requires Rho GTPase (Dupont et al., 2011); 5) Contact inhibition which will change the F-actin arrangement leads to the inactivation of TAZ (Lei et al., 2008; Liu et al., 2010). Actin cytoskeleton appears to be the master regulator of the Hippo-YAP/TAZ pathway, and multiple extracellular signals are integrated to the cytoskeleton reorganization to transduce the signals to the core of the Hippo kinases module. However, how the cytoskeleton arrangement is sensed by the Hippo pathway remains elusive. Another interesting observation is that the F-actin arrangement is tightly regulated by TAZ during EMT processes (Lei et al., 2008; Liu et al., 2010). For the extensive discussion and implication of actin cytoskeleton and the Hippo signaling pathway, please refer to other insightful reviews (Gaspar & Tapon, 2014; Matsui & Lai, 2013; Yu & Guan, 2013).

### Ligands to GPCRs

Recently, a link between extracellular ligands and the Hippo pathway has been identified (Miller et al., 2012; Yu et al., 2012) (Fig. 2). Under serum deprivation condition, LATS1/2 are activated independent of MST1/2 and thereby phosphorylate and inactivate YAP/TAZ. Treatment with lysophosphatidic acid (LPA) and sphingosine-1-phosphate (S1P), two major lipids in serum, results in the dephosphorylation and accumulation of TAZ via the corresponding GPCRs and the cognate Gα12/13 (Miller et al., 2012; Yu et al., 2012). Consistent with the role of LPA and S1P in modulating TAZ, Zhou et al. demonstrated that estrogen could also induce the activation of TAZ in multiple breast cancer cells. Stimulation of GPER, the G protein-coupled estrogen receptor, activates Gαq/11, PLCβ-PKC, and Rho-ROCK to inactivate LATS1/2, leading to the dephosphorylation and accumulation of TAZ. More importantly, total TAZ level and nuclear TAZ correlate with GPER expression in human invasive ductal breast cancer specimens (Zhou et al., 2015a). Mo et al. also proved that thrombin, the ligand of protease-activating receptors (PAR1) could induce dephosphorylation and activation of TAZ in a similar mechanism (Mo et al., 2012). In contrast to Gα12/13, Gαq/11, and Gαi/o, Gαs plays an opposing role on TAZ activation. Yu et al. found that overexpression of Gαs induces LATS1/2 activation and TAZ phosphorylation (Yu et al., 2012). The ligands, such as glucagon and dobutamine which can stimulate Gαs, indeed abolish the activation of TAZ (Bao et al., 2011; Yu et al., 2012). Cyclic adenosine monophosphate (cAMP), a second messenger downstream of Gαs-couple receptors, mediates Gαs-induced TAZ inactivation via PKA and Rho GTPase (Yu et al., 2013). GPCRs represent the largest family of membrane receptors in mammals and mediate numerous signals during physiological and pathological conditions. The link between GPCRs and the Hippo signaling pathway provides novel insights into the regulation of TAZ, implying the multiple functions of TAZ in different cell types and different circumstances (Yu & Guan, 2013; Zhou et al., 2015b). G proteins and GPCRs are frequently altered in human cancers (Kan et al., 2010; O’Hayre et al., 2013). Recently, two groups found that around 80% of uveal melanoma harbor *GNAQ* or *GNA11* mutation and YAP/TAZ are constitutively stimulated in these specimens (Feng et al., 2014; Yu et al., 2014). Verteporfin (VP), an inhibitor of YAP/TAZ-TEADs interaction, blocks the tumor growth of uveal melanomas carrying mutations in *GNAQ* and *GNA11*. These studies add the possibility of using YAP/TAZ as therapeutic targets for cancer treatment. We look forward to seeing more follow-up studies on GPCRs and the Hippo-YAP/TAZ pathway, which would uncover the multiple regulations on this exciting pathway and accelerate the discovery of new cancer treatment approaches.

### Metabolism and nutrient

Metabolism switch is one of the hallmarks of cancer (Hanahan & Weinberg, 2011). Recent studies have implicated that energy status, glycolysis and mevalonate biosynthesis could modulate YAP/TAZ activities (Fig. 2). These studies suggest that TAZ could response to and coordinate with the nutrient status to regulate gene transcription and cell proliferation.

Glucose metabolism is a central metabolism pathway to produce energy and carbon for the building blocks of cellular synthesis. Metabolic reprogramming from oxidative respiration to aerobic glycolysis commonly happens in cancer cells, which is called Warburg effect (Hanahan & Weinberg, 2011; Warburg, 1956a; Warburg, 1956b). Growing evidence suggests that the aerobic glycolysis can support the oncogenic signaling to foster tumor malignancy. Recent studies connected the energy status to the Hippo-YAP/TAZ pathway (Mo et al., 2015; Wang et al., 2015b). Under glucose deprivation, LATS1/2 are strongly activated and thereby inhibit TAZ (Mo et al., 2015). AMPK, one of the major kinases sensing glucose, phosphorylates AMOTL1 and hence activates LATS1/2 (DeRan et al., 2014). A second proposed model is that AMPK can phosphorylate YAP directly at serine 94 which is required for its interaction with TEADs (Mo et al., 2015; Wang et al., 2015a). However, whether this mechanism is conserved in the regulation of TAZ by energy status remains unclear. Glucose could activate YAP/TAZ in a way independent of AMPK and LATS1/2 (Enzo et al., 2015). When glucose uptake or a shift from glycolysis to oxidative phosphorylation is blocked, the transcriptional activities of YAP/TAZ are decreased via modulating the complex formation between TEADs and YAP/TAZ (Enzo et al., 2015). Mechanistically, they showed that PFK1 (phosphofructokinase), the enzyme of the first committed step of glycolysis, interacts with TEADs, and the transcriptional coactivation activity of TAZ is reduced when *PFK1* is knocked-down. (Enzo et al., 2015). Moreover, in a large dataset of primary human mammary tumors, YAP/TAZ activities are increased in high-grade (G3 vs. G1) tumors and strongly associated with genes regulated by glucose metabolism and breast cancer malignancy (Conley et al., 2012; Schwab et al., 2012). All these findings suggest that the activity of TAZ is tightly modulated by glucose uptake or glycolysis, and TAZ is one of the key downstream effects mediating the transcriptional reprogramming upon glucose status fluctuation. Under glucose crisis, TAZ is restricted to prevent further exhaustion to maintain cell survive.

Coincidently, Sorrentino et al. screened a library containing 650 FDA-approved compounds and found that statins which are inhibitors of HMGCR (HMG-CoA reductase) could profoundly decrease YAP/TAZ nuclear localization (Sorrentino et al., 2014). Mechanistically, HMGCR is the rate-limiting enzyme of the mevalonate pathway which produces geranylgeranyl pyrophosphate (GGPP) for prenylation and membrane association of Rho GTPase, and it has been well-established that Rho GTPase can act as an activator of TAZ (Dupont et al., 2011; Yu et al., 2012; Zhao et al., 2012). Furthermore, inhibition of geranylgeranyl transferase, another enzyme of the mevalonate pathway, by GGTI-2133 impressively reduced the activity of YAP (Wang et al., 2014). Although Wang et al. didn’t test GGTI-2133’s effect on TAZ, it should have a similar impact as on YAP. It makes sense that perturbing Rho GTPase by these compounds is sufficient to modulate TAZ activity because Rho/ROCK/F-actin plays a central role in regulating the Hippo-YAP/TAZ pathway. Screening or modifying the compounds targeting the mevalonate pathway would be one direction to inhibit the oncogenic roles of TAZ in human cancer.

### Hypoxia

Hypoxia is one of the crucial microenvironmental factors that promote tumorigenesis (Vaupel & Mayer, 2007; Wilson & Hay, 2011). Hypoxia has also been shown to induce the tumor-initiating activity of cancer stem cells in breast cancer through regulating the activity of hypoxia-inducible factors (HIFs) (Conley et al., 2012; Schwab et al., 2012). Recent studies imply that there is a bidirectional crosstalk between HIF-1 and TAZ to co-regulate gene transcription under hypoxia environment. Xiang et al. reported that the TAZ is regulated by hypoxia condition to induce breast cancer stem cell phenotype in two discrete mechanisms (Xiang et al., 2014). First, the mRNA level of TAZ and its target genes are upregulated under hypoxia condition (1% O_2_) in multiple breast cancer cell lines, and this phenomenon is dependent on HIF-1. They further confirmed that HIF-1 binds directly to the HRE (HIF response element) which locates between exon 2 and exon 3 of *WWTR1* gene under hypoxia condition, and promotes the transcription of TAZ; second, the authors found that there is more nuclear TAZ under hypoxia condition. Mechanistically, they observed that the proteasome degradation of LATS2, which is the upstream kinase of TAZ in the Hippo signaling pathway, is induced by hypoxia in an HIF-1 and SIAH1 dependent manner (Xiang et al., 2014). Moreover, based on the analysis of human breast cancer database, the author found that only simultaneous TAZ^high^ and HIF-1^high^ expression status is correlated with worse survival. Consistently, Yan et al. found that both the hypoxia condition (1% O_2_) and hypoxia mimics (DMOG and CoCl2) promote TAZ expression via an HIF-1α-dependent manner in ovarian cancer cells (Yan et al., 2014). Interestingly, the follow-up study from Xiang and colleagues demonstrate that TAZ and HIF-1α interact with each other and functionally serve as reciprocal transcriptional co-factors. HIF-1α serves as a coactivator of TAZ/TEADs complex for the transcription of target genes (such as *CTGF*) and TAZ serves as a coactivator of HIF-1α for the transcription of target genes (such as *PDK1* and *LDHA*) in hypoxic human breast cancer cells. Either knockdown of *TAZ* or *HIF-1α* decreases the enrichment of HIF1 or TAZ in HRE of *PDK1* (Xiang et al., 2015). One consistent observation was found by Bendinelli and colleagues (Bendinelli et al., 2013). They showed that HIF-1α and TAZ interact with each other and are co-localized in the nucleus after hypoxia. More importantly, the activity of HIF-1 is tightly regulated by TAZ, indicating that TAZ may be involved in the bone metastasis of breast cancer under hypoxic microenvironment (Bendinelli et al., 2013). The crosstalk between HIF-1 and TAZ increases the transcriptional activity of both pathways and contributes to the gene expression under hypoxic microenvironment. Therapeutic strategies that inhibit HIF-1α or TAZ or combination of both may improve the cancer treatment, especially for solid tumors that suffer from severe hypoxic condition inside the tumors.

### Osmotic stress

TAZ is highly expressed in kidney, and *TAZ* knockout mice develop multiple renal cysts and urinary concentration defects (Hossain et al., 2007; Makita et al., 2008). However, the molecular mechanism of how TAZ functions in renal cells remains elusive. A recent study showed that hyperosmotic stress selectively enhanced the phosphorylation of TAZ at tyrosine 316 via c-Abl activation, and this site-specific phosphorylation is required for TAZ interaction with NFAT5 in response to osmotic stress. Moreover, the phosphorylated TAZ suppressed the DNA-binding and transcriptional activity of NFAT5 (Jang et al., 2012). This finding raised an intriguing hypothesis that TAZ may work as a transcriptional co-repressor in renal cells under hypertonic conditions, and the physiological significance of this phenomenon remains to be explored. As we know, serine 89 is the major phosphorylated site in TAZ by LATS1/2, and phosphorylation at this site leads to the cytoplasm retardation and inactivation of TAZ (Huang et al., 2012; Lei et al., 2008; Liu et al., 2010). Therefore, it’s interesting to test whether the phosphorylation status of serine 89 in TAZ is altered upon osmotic stress. In contrast, Jung-Soon Mo et al. showed that osmotic stress cannot induce TAZ phosphorylation using a phos-tag gel in HEK293A cells (Mo et al., 2015). It should be noticed that these two groups use different cell lines and different methods to induce osmotic stress, and these may be the reasons for the controversial results.

## **TAZ AND HUMAN CANCERS**

In addition to the work done in cell culture, increasing numbers of studies have shown that TAZ are elevated or activated in multiple human cancers, including breast cancer, glioblastoma, lung cancer, colorectal cancer, and oral squamous cell carcinoma (See discussion below).

### Breast cancer

TAZ protein level and activity are up-regulated in high-grade metastatic breast cancers, and overexpression of TAZ is sufficient to induce breast cancer cell proliferation, transformation, and EMT (Chan et al., 2008; Cordenonsi et al., 2011; Lei et al., 2008). Furthermore, TAZ is required for and sufficient to maintain self-renewal and tumor initiation capability of breast cancer stem cells (Cordenonsi et al., 2011). Bartucci and colleagues provide evidence that TAZ is required for metastatic activity and chemoresistance of breast cancer stem cells, and TAZ expression level negatively correlates with shorter disease-free survival of breast cancer patients (Bartucci et al., 2015). TAZ also serves as a biomarker for decreased pathological complete response rate in luminal B/HER2-positive breast cancer patients who received neoadjuvant trastuzumab or chemotherapy (Vici et al., 2014). TAZ expression level is significantly correlated with GPER, G protein-coupled receptor of estrogen, in human invasive ductal breast cancer, and may contribute to tamoxifen resistance of breast cancer therapy (Zhou et al., 2015a). Extensive studies should focus on the mechanism of the oncogenic role of TAZ in breast cancer development using mouse model.

### GBM (Glioblastoma multiforme)

Compared with the proneural (PN) GBMs and low-grade gliomas, mesenchymal (MES) GBMs have elevated TAZ expression due to the lower methylation level in its promoter region (Bhat et al., 2011). Actually, TAZ could be recruited to numerous mesenchymal gene promoters and drives the mesenchymal feature of malignant glioma (Bhat et al., 2011). Resistance to Temozolomide, one commonly used chemotherapy drug for GBM, is frequently happened (Hegi et al., 2005). Tian et al., showed that TAZ promotes temozolomide resistance by reducing temozolomide-induced apoptosis. High level of TAZ expression predicts a poor outcome of GBM patients (Tian et al., 2015). Glioma cancer stem cells (GSCs) are one of the major reasons for chemotherapy resistance in GBM patients (Bao et al., 2006). Since we have already known that TAZ is required for and sufficient to maintain self-renewal and tumor initiation capability of breast cancer stem cells (Cordenonsi et al., 2011), it’s interesting to study whether TAZ-induced temozolomide resistance is related to TAZ’s function in GSCs.

### Lung adenocarcinoma

TAZ was firstly identified as an oncogene in non-small cell lung cancer (NSCLC) in 2011 (Zhou et al., 2011). TAZ expression is a prognostic indicator for worse survival in resected NSCLC (Noguchi et al., 2014; Xie et al., 2012). Knockdown of *TAZ* in NSCLC cell lines is sufficient to suppress proliferation, invasion, and tumor growth (Wang et al., 2010; Zhou et al., 2011). Additionally, a YAP/TAZ gene expression signature is significantly associated with tumor-propagating cells and human lung cancer progression (Lau et al., 2014). A higher level of TAZ indicates worse overall survival and more frequent metastasis in lung adenocarcinoma patients (Lau et al., 2014). The highly expressed TAZ in NSCLC may also be on the reasons for gefitinib resistance in cells harboring EGFR-T790M mutation (Xu et al., 2015). It’s worthy to study TAZ’s function in lung cancer systemically because *TAZ* knockout mice develop multiple renal cysts and lung emphysema (Hossain et al., 2007; Makita et al., 2008).

### Colorectal cancer

Recent two studies provided evidence that TAZ expression is an independent prognostic indicator in colorectal cancer (Wang et al., 2013; Yuen et al., 2013). Colorectal cancer patients carrying co-overexpression of YAP and TAZ have a worse outcome than those who have either one alone (Wang et al., 2013). Knockdown of *YAP* and *TAZ* in colorectal cancer cells reduces the proliferation, metastasis, and invasion (Wang et al., 2013).

### Oral squamous cell carcinoma

Several studies observed that the expression of TAZ in tongue squamous cell carcinoma significantly correlated with tumor size, pathological grade, and clinical stage. Higher expression of TAZ is negatively associated with overall survival and disease-free survival (Hiemer et al., 2015; Li et al., 2015b; Wei et al., 2013). Notably, TAZ is also required for the maintenance of self-renewal and tumor initiation ability of oral cancer stem cells (Li et al., 2015b).

### Kaposi sarcoma

A recent study demonstrated that KS-associated herpesvirus (KSHV), a GPCR, activates Gαq/11 and Gα12/13 to inhibit LATS1/2 and thereby dephosphorylates and activates TAZ (Liu et al., 2015). They also showed that the expression level of TAZ is elevated in human Kaposi sarcoma specimens (Liu et al., 2015).

### Epithelioid hemangioendothelioma

Based on the documents and TCGA database, rare mutations of TAZ were observed in human cancer specimens (Harvey et al., 2013; Yu et al., 2015). Recently, chromosomal translocation of TAZ was found in a rare vascular sarcoma termed epithelioid hemangioendothelioma. Remarkably, gene fusion of *WWTR1-CAMTA1* (calmodulin-binding transcription activator 1) happens in virtually all epithelioid hemangioendothelioma (Errani et al., 2011; Tanas et al., 2011). Mechanistically, TAZ-CAMTA1 fusion results in nuclear localization and constitutive activation of TAZ (Tanas et al., 2016).

As we discussed above, overexpression or hyperactivation of TAZ is widespread in human cancers, indicating that TAZ is important for the development and sustainability of neoplasia. Actually, *in vitro* cell culture studies showed that either gain or loss of TAZ can enhance or suppress cancerous phenotypes in a wide range of cell lines. Consistent with the observation that TAZ is highly expressed in multiple human cancers, TAZ displays the transforming properties in cell culture system. Over-expression of TAZ in cultured cells leads to cancer features such as anchorage-independent growth (Chan et al., 2011; Yang et al., 2012), epithelial to mesenchymal transition (EMT) (Chan et al., 2008; Cordenonsi et al., 2011; Hong et al., 2005; Lei et al., 2008), growth-factor-independent proliferation (Yang et al., 2012), resistance to chemotherapeutics (Xu et al., 2015), increased migration, invasion, tumor-initiation properties, and tumor formation in xenograft models (Bartucci et al., 2015; Cordenonsi et al., 2011; Lau et al., 2014; Yuen et al., 2013). In addition, TAZ endows self-renewal capability of breast cancer cells to sustain the cancer stem cells population (Cordenonsi et al., 2011). Concordantly, loss of TAZ inhibits cancerous phenotypes in cancer cell lines (Bartucci et al., 2015; Cordenonsi et al., 2011; Lau et al., 2014; Yuen et al., 2013; Zanconato et al., 2015). For example, the capability of anchorage-independent growth, migration, invasion, and tumorigenesis of MCF7 breast cancer cells is decreased when *TAZ* is knocked-down (Chan et al., 2008). Likewise, siRNA knockdown of *TAZ* in A549 cells inhibits anchorage-independent growth and tumor growth in mice (Zhou et al., 2011). Collectively, TAZ plays an important role in tumorigenesis via regulating multiple aspects of cancer cells, implying that TAZ could be served as a nice candidate for cancer diagnosis or therapy.

## TARGETING TAZ FOR CANCER THERAPY

Accumulating studies provide evidence that TAZ is an oncoprotein during tumorigenesis, leading us to consider the therapeutic benefits of TAZ inhibition in cancer (Bhat et al., 2011; Chan et al., 2008; Zhang et al., 2009). Kinases always serve as the best targets for small-molecular inhibitors. However, unlike most oncogenic kinases in cancer, the kinases in the Hippo pathway are tumor suppressors. This means it is unlikely to kill the cancer cells if we target the kinases in the Hippo pathway by small-molecular inhibitors. Other ways should be explored beyond targeting the core kinases module.

The major readout of the Hippo-YAP/TAZ pathway is modulating the downstream target gene transcription via interacting with and coactivating TEADs transcription factors (Zhang et al., 2009). Compounds which disrupt the interaction between TAZ-TEADs are considered as good candidates for inhibition of the oncogenic roles of TAZ (Sudol et al., 2012). Liu-Chittenden et al. screened around 3000 compounds and found that members of the porphyrin family, such as verteporfin (VP), hematoporphyrin (HP), and protoporphyrin IX (PPIX), are strong candidates of YAP/TAZ inhibitors (Liu-Chittenden et al., 2012). Verteporfin, which is a clinical photosensitizer in photocoagulation therapy for macular degeneration, strikingly dissociates the interaction between YAP/TAZ and TEADs and thereby inhibits the downstream target genes transcription. More importantly, verteporfin blocked YAP-induced liver tumorigenesis and also exhibits an anti-cancer effect on uveal melanoma cells carrying *GNAQ* mutations (Liu-Chittenden et al., 2012). However, we couldn’t exclude the possibility that verteporfin can inhibit cancer cell proliferation and induce cancer cell death in a way independent of YAP/TAZ (Zhang et al., 2015a). Recent studies showed that VGLL4 is a novel negative regulator of YAP-TEADs complex, and a peptide mimicking VGLL4 suppressed tumor growth of human primary gastric cancer in nude mice (Jiao et al., 2014; Zhang et al., 2014). Based on the structure of the YAP/TEAD complex, VGLL4 should display a similar effect on disrupting the interaction between TAZ and TEADs.

Another possibility of abolishing TAZ activity is disturbing the upstream regulators. As we discussed above, Rho GTPase and actin cytoskeleton exhibit central role in the regulation of the Hippo-YAP/TAZ in response to a wide range of upstream signals (Yu et al., 2015). Hence, inhibition of Rho GTPase would activate LAT1/2 kinase activity and thereby inhibit TAZ. Recently, Sorrentino et al. found that statins, inhibitors of HMGCR which is the rate-limiting enzyme of the mevalonate pathway, could strongly reduce YAP/TAZ nuclear localization (Sorrentino et al., 2014). The mevalonate pathway is essential for prenylation, membrane association, and activation of Rho GTPase. Inhibition of HMGCR strongly activates LATS1/2 and reduces the transcriptional activity of TAZ, hence exhibiting anti-proliferation and apoptotic effects on breast cancer (Sorrentino et al., 2014). It’s intriguing to test whether other clinically used inhibitors of the mevalonate pathway, such as zoledronic acid (FDPS inhibitor), GGTI-2133 (GGPP inhibitor), and fatostatin (SREBP inhibitor), have a similar effect as statins.

The above studies clearly indicate a powerful therapeutic capacity of targeting TAZ in human cancer. Further investigation of the regulation and function of TAZ will help us to uncover the mystery of this amazing protein and apply these finding to cancer therapy.

## **OUTSTANDING QUESTIONS**

Since TAZ was firstly identified as a 14-3-3 binding protein in 2000, rapid research progress has been achieved for a better understanding of TAZ. However, some key questions remain unanswered, and new questions arise. Here we listed below some of the key questions that should be addressed.

(1) What’s the difference between TAZ and YAP? TAZ and YAP contain similar domains/motifs, including 14-3-3 binding motif, WW domain, coiled-coil domain, and a PDZ-binding motif. As downstream effectors of the Hippo signaling pathway, YAP and TAZ are also similarly regulated. However, these two proteins have distinct tissue expression pattern (Kanai et al., 2000; Sudol et al., 1995), indicating different functions in specific organs.

(2) Are there any other regulatory posttranslational modifications happening on TAZ beside phosphorylation and polyubiquitylation? TAZ can be phosphorylated by LATS1/2, GSK3, CK1, c-Abl, and Cdk1, and these phosphorylated sites are involved in the regulation of subcellular localization, stability, and activity of TAZ. Other modifications on TAZ may provide novel insight into the regulation and function of TAZ.

(3) How does cytoskeleton dynamic regulate LATS1/2 activity? Increasing evidence points out that cytoskeleton remodeling plays a central role in the regulation of the Hippo pathway in response to multiple intrinsic and extracellular signals. However, the kinase which senses the signal from cytoskeleton remodeling and then phosphorylates LATS1/2 remains elusive.

(4) How could we utilize TAZ as a therapeutic target in human cancer? To answer this question, we should study the regulation and function of TAZ extensively in cancer cells, especially using the mouse cancer model. Accumulating studies suggest that TAZ could be regulated by a broad range of signals, leading us to reconsider the way we could use to modulate the activity of TAZ in cancers with high expression level of TAZ.
